# TLR4/NF-κB-Mediated Anti-Inflammatory and Cognitive Protective Actions of *Curcuma longa* and Ar-turmerone in Alzheimer’s Disease Models

**DOI:** 10.4014/jmb.2506.06044

**Published:** 2025-09-17

**Authors:** Kyo-Nyeo Oh, Donghyuck Bae, Dool-Ri Oh, Ji-Ae Hong, Yujin Kim, Eun Kim, Seon ah Son, Kwang Youl Lee, Sunoh Kim

**Affiliations:** 1 Jeonnam Bio Foundation, Green Bio Headquarters, Natural Resources Laboratory, Jangheung-gun, Jeollanamdo 59338, Republic of Korea; 2 Central R&D Center, B&Tech Co., Ltd., Naju 58205, Republic of Korea; 3 Research Institute of Pharmaceutical Sciences, College of Pharmacy, Chonnam National University, Gwangju 61186, Republic of Korea

**Keywords:** *Curcuma longa*, ar-turmerone, Alzheimer’s disease, TLR4, NF-κB, amyloid beta

## Abstract

In our previous study, we systematically compared various extraction methods and identified the 80% ethanolic extract of *Curcuma longa* L. (CL-80) as the most effective in protecting neurons from stress-induced damage in both *in vitro* and *in vivo* models. Although curcumin, a major constituent of *C. longa*, has demonstrated neuroprotective effects, the role of ar-turmerone, a bioactive sesquiterpenoid also derived from *C. longa*, remains underexplored in Alzheimer’s disease (AD) models. In this study, we evaluated the anti-inflammatory and cognitive-protective effects of CL-80 and ar-turmerone in both *in vitro* and *in vivo* models of amyloid-β (Aβ)-induced neurotoxicity. Primary cultured rat hippocampal neurons exposed to Aβ_25−35_ showed significantly increased expression of TNF-α, IFN-β, and iNOS, all of which were dose-dependently attenuated by CL-80 or ar-turmerone. Furthermore, both compounds suppressed Aβ-induced activation of the TLR4/NF-κB signaling axis at mRNA and protein levels. In an Aβ_1−42_-injected mouse model, oral administration of CL-80 or ar-turmerone significantly improved learning and memory performance in the Morris water maze and passive avoidance tests. Biochemical analyses of hippocampal tissues revealed reduced TLR4 expression and NF-κB activation, decreased acetylcholinesterase (AChE) activity, and restoration of acetylcholine (ACh) levels following treatment. Collectively, these findings suggest that both CL-80 and ar-turmerone exert neuroprotective effects by inhibiting TLR4/NF-κB-mediated neuroinflammation and preserving cholinergic function in an AD animal model. This study offers novel insight into the therapeutic potential of *C. longa* constituents for the treatment of AD.

## Introduction

Alzheimer’s disease (AD) is a progressive neurodegenerative disorder characterized by the accumulation of extracellular amyloid-beta (Aβ) plaques, intracellular neurofibrillary tangles, neuronal loss, and activation of microglia in the brain [1, 2]. Aβ plays a pivotal role in AD pathogenesis by inducing oxidative stress and activating the innate immune response through microglial stimulation [3-5]. Accordingly, numerous therapeutic strategies have been developed to reduce Aβ deposition and mitigate its downstream inflammatory effects within the central nervous system (CNS) [6]. Among these pathological processes, neuroinflammation has emerged as a critical hallmark of AD progression [7, 8], with elevated levels of pro-inflammatory mediators such as cytokines, chemokines, acute-phase proteins, and complement components observed in AD brains [9].

Aβ aggregation is closely associated with microglial activation and subsequent release of inflammatory cytokines [10]. Toll-like receptors (TLRs), a family of transmembrane pattern-recognition receptors, are central mediators of innate immune responses. Among the 10 TLRs identified in humans (TLR1–TLR10), TLR4 has been particularly implicated in AD pathophysiology due to its capacity to recognize Aβ and initiate downstream signaling cascades involving nuclear factor-kappa B (NF-κB), ultimately leading to pro-inflammatory gene expression [11, 12]. Increased expression of TLR2 and TLR4, along with elevated NF-κB activity, has been observed in postmortem AD brains and Aβ-stimulated animal models [13-15]. Moreover, TLR4 mutant mice exhibit attenuated tumor necrosis factor-α (TNF-α) expression following Aβ exposure compared to wild-type controls, suggesting that microglia-mediated cytokine release in AD is at least partly TLR-dependent [3, 16]. Despite accumulating evidence implicating TLR4/NF-κB signaling in AD-related neuroinflammation and neuronal degeneration, the specificity of this pathway in regulating microglia-mediated neuronal cell death remains incompletely understood.

*Curcuma longa* L. (turmeric) has long been utilized in traditional medicine for its wide-ranging pharmacological properties, including anti-inflammatory [17], antioxidant [18], anticancer [19], antimicrobial [20], and antidepressant activities [21]. Turmeric extracts and their bioactive compounds have been reported to inhibit Aβ formation both *in vitro* and *in vivo* [22], positioning *C. longa* as a promising candidate for the development of AD therapeutics [23]. Curcumin, the most extensively studied polyphenol in *C. longa*, exerts neuroprotective effects via modulation of inflammatory cytokines, oxidative stress pathways, and transcription factors such as NF-κB and activator protein 1 (AP-1) [24, 25]. In preclinical AD models, curcumin has been shown to attenuate cognitive deficits and suppress neuroinflammatory responses [26, 27]. Its ability to modulate both intracellular and extracellular pathways involved in Aβ production and aggregation has been well documented [28, 29]. In contrast, aromatic-turmerone (ar-turmerone), a bisabolene-type sesquiterpenoid and a major volatile component of turmeric essential oil, has only recently garnered scientific interest for its actions within the CNS [30, 31]. Structurally, ar-turmerone contains a lipophilic aromatic ring with a ketone side chain, allowing it to penetrate the blood-brain barrier (BBB) and persist in brain tissue, features favorable for CNS-targeted therapy [32-34]. Ar-turmerone has been shown to exert anticonvulsant effects and modulate neuronal gene expression related to plasticity and excitability, including c-fos and brain-derived neurotrophic factor (BDNF), without inducing motor impairment in animal models [35, 36]. Moreover, ar-turmerone has demonstrated anti-inflammatory potential by downregulating pro-inflammatory mediators and suppressing microglial activation, particularly in response to Aβ or lipopolysaccharide (LPS) stimulation [37]. These actions are presumed to involve inhibition of NF-κB, c-Jun N-terminal kinase (JNK), and p38 mitogen-activated protein kinases (p38 MAPK) signaling pathways. However, most of these findings are limited to *in vitro* studies using microglial or primary neuronal cultures, and there remains a lack of *in vivo* evidence validating its efficacy in AD models. Given the emerging role of TLR4/NF-κB signaling in Aβ-induced neuroinflammation, it is crucial to investigate whether ar-turmerone can modulate this pathway in physiologically relevant AD animal models.

Thus, the present study aimed to evaluate the effects of *C. longa* extract and ar-turmerone on Aβ-induced neuroinflammatory responses, with particular emphasis on the TLR4/NF-κB signaling pathway. Using both *in vitro* and *in vivo* AD models, we sought to determine whether these compounds could attenuate neuroinflammation and cognitive impairment through suppression of this pathway, thereby providing new insight into their therapeutic potential for AD.

## Materials and Methods

### Reagents

Aβ_1-42_, Aβ_25-35_, and acetylcholine (ACh) were purchased from Sigma-Aldrich (USA), and the Cell Death Detection Kit was obtained from Roche Diagnostics GmbH (Germany). Monoclonal antibodies against TLR4, NF-κB, and β-actin, as well as horseradish peroxidase (HRP)-conjugated secondary antibodies, were acquired from Santa Cruz Biotechnology Inc. (USA). ELISA kits for TNF-α, interferon beta (IFN-β), and inducible nitric oxide synthase (iNOS) were obtained from R&D Systems Inc. (USA). Trypsin, neurobasal medium, and B27 supplement were purchased from Thermo Fisher Scientific Inc. (Gibco, Germany). All other reagents used were of analytical grade.

### Preparation of 80% Ethanol Extract (CL-80), Fractionation, and Isolation of ar-Turmerone from *Curcuma longa* L.

*C. longa* was collected from Jindo Island, Jeollanam-do, South Korea, in 2022 and authenticated by Dr. Kim at B&Tech Co. Ltd., (Republic of Korea). The extraction protocol followed our previously reported method [38]. Briefly, dried *C. longa* rhizomes were ground and extracted with 20 volumes of 80% ethanol (*v/w*) at 75°C for 4 h. The extract was filtered, and the filtrate was evaporated under reduced pressure. The resulting concentrate was freeze-dried to obtain a yellow-brown powder, designated CL-80. The dry matter content was determined by drying at 105°C to a constant weight (Table 1). CL-80 (204 g) was suspended in distilled water and successively partitioned with *n*-hexane, chloroform (CHCl_3_), ethyl acetate (EtOAc), and *n*-butanol (*n*-BuOH), yielding four solvent fractions: *n*-hexane (38.3 g), CHCl_3_ (75.1 g), EtOAc (13.6 g), *n*-BuOH (25.4 g) and aqueous fraction (51.6 g). The *n*-hexane-soluble fraction was further subjected to silica gel column chromatography using an *n*-hexane:acetone gradient (from 100:0 to 1:1, *v/v*), and subdivided into four subfractions (CH-1 to CH-4). Each fraction was concentrated under reduced pressure, freeze-dried, and stored at 4°C until further use.

### High-Performance liquid chromatography (HPLC) Analysis of ar-Turmerone

HPLC was conducted using a diode array detector (DAD) set at 425 nm to quantify the ar-turmerone content. Standard ar-turmerone was employed for calibration, and the retention times were compared with those of the sample fractions (CH-1–CH-4). The chromatographic analysis revealed a distinct peak corresponding to ar-turmerone in the CH-2 fraction, exhibiting strong UV absorbance at 425 nm. All measurements were performed in triplicate to ensure reproducibility.

### Animals

Male ICR mice (18–20 g) and time-pregnant Sprague-Dawley (SD) rats at full-term gestation were purchased from Central Lab Animal Inc., (Republic of Korea). All animals were housed under controlled environmental conditions: temperature of 22 ± 2°C, relative humidity of 50 ± 5%, and a 12:12 h light:dark cycle (lights on at 8:00 a.m.), with free access to standard chow and water. All experimental procedures were conducted in accordance with institutional guidelines for the care and use of laboratory animals and were approved by the Institutional Animal Care and Use Committee (IACUC) of Bioresources and Technology (B&Tech) Co. Ltd., (Republic of Korea; Approval No. BT-003-2022).

### Primary Culture of Rat Hippocampal Neurons and Treatments

Primary hippocampal neuron cultures were prepared as previously described [39]. Briefly, hippocampi were isolated from embryonic day 18 (E18) SD rat embryos and dissociated using 0.25% trypsin in Hank's Balanced Salt Solution (HBSS). The cells were seeded in neurobasal medium supplemented with B27 and incubated at 37°C in a humidified atmosphere of 5% CO_2_. Experiments were conducted on neurons at day 10 *in vitro* (DIV 10). Cells were treated with or without Aβ_25-35_ (50 μM) at a density of 1 × 10^5^ cells/ml, followed by administration of CL-80 or ar-turmerone at various concentrations.

### Neuronal Viability Assay

Neuronal viability was assessed by measuring the dehydrogenase activity of living cells using a standard 3-(4,5-dimethylthiazol-2-yl)-2,5-diphenyltetrazolium bromide (MTT) assay. Cultured hippocampal neurons were washed with neurobasal medium lacking B27 supplement and incubated with Aβ_25-35_ (50 μM) alone or in combination with test compounds for designated time periods. Following treatment, MTT solution (5 mg/ml, 50 μl/well) was added, and the cells were incubated for 4 h at 37°C. After incubation, the supernatants were carefully removed, and the resulting formazan crystals were solubilized in 100 μl of DMSO. The optical density was measured at 540 nm using a microplate reader (BioTek, USA).

### Aβ_1−42_-Induced Alzheimer's Disease (AD) Mouse Model

Mice were deeply anesthetized with pentobarbital (40 mg/kg, intraperitoneally) and placed in a stereotaxic apparatus (Harvard Apparatus, USA). Aggregated Aβ_1-42_ peptide (4 μg in 1 μl) was unilaterally injected into the hippocampal region at the following coordinates: anteroposterior (AP) −3.6 mm, mediolateral (ML) +2.4 mm, and dorsoventral (DV) −2.8 mm, using a microsyringe at a rate of 1 μl/min. After surgery, each mouse received an intramuscular injection of penicillin (100,000 U) into the hindlimb and was housed individually. Behavioral assessments were initiated two days after the injection.

### Experimental Groups and Administration

Forty-nine naïve mice were randomly assigned to seven groups (*n* = 7 per group) as follows:

(1) vehicle-injected and orally administered 1% Tween 20 (Control group); (2) Aβ_1-42_-injected and orally administered 1% Tween 20 (Aβ_1-42_ group); (3) Aβ_1-42_-injected and treated with silk fibroin hydrolysate (SFH, Shindo Biosilk, Republic of Korea) at 100 mg/kg (SFH 100 group, positive control); (4) Aβ_1-42_-injected and treated with CL-80 at 100 mg/kg (CL-80 100 group); (5) Aβ_1-42_-injected and treated with CL-80 at 300 mg/kg (CL-80 300 group); (6) Aβ_1-42_-injected and treated with ar-turmerone at 5 mg/kg (ar-turmerone 5 group); (7) Aβ_1-42_-injected and treated with ar-turmerone at 10 mg/kg (ar-turmerone 10 group). This administration schedule was designed to assess the preventive efficacy of CL-80 and ar-turmerone against Aβ-induced neuropathology, by priming the brain environment prior to Aβ_1-42_ injection. All test compounds (SFH, CL-80, and ar-turmerone) were dissolved in 1% Tween 20 and administered orally once daily at 9:00 a.m. for 21 consecutive days prior to Aβ_1-42_ hippocampal injection, and continued for an additional 7 days post-injection. The doses of ar-turmerone (5 and 10 mg/kg) were selected based on previous studies demonstrating neuroprotective efficacy and BBB permeability in rodents conducted to determine the minimum effective dose [35, 36].

### Morris Water Maze – Learning and Memory Assessment

Spatial learning and memory were evaluated using the Morris water maze with minor modifications from the original protocol [40]. The apparatus consisted of a circular pool (diameter: 100 cm; height: 45 cm) filled with water to a depth of 26 ± 1.5 cm. To render the water opaque, non-fat milk was added. A transparent escape platform (height: 25 cm) was submerged approximately 1 cm below the water surface and remained in a fixed location throughout the training period. On day 1, mice underwent a pre-training session without the platform and were allowed to swim freely for 1 min. From day 2 to day 4, mice were subjected to four training trials per day with inter-trial intervals of 5 min. In each trial, mice were released from one of four pseudo-randomly assigned starting points. The latency to locate the hidden platform was recorded as a measure of spatial learning. On day 5 (probe trial), the platform was removed to assess memory retention. Each mouse was allowed to swim freely for 90 s, and the amount of time spent in each quadrant of the pool was recorded. Data were analyzed using a video tracking system (SMART Video Tracking System, USA).

### Passive Avoidance Test

One-trial step-through passive avoidance learning was assessed using a commercially available apparatus (Iwoo Scientific Co., Republic of Korea), consisting of a brightly lit chamber and a dark chamber separated by a guillotine door. The dark chamber featured a stainless-steel grid floor capable of delivering a mild foot shock. During the acquisition trial, each mouse was placed in the light chamber for a 1-min habituation period. The guillotine door was then opened to allow the mouse to enter the dark chamber. Upon full entry, the door was closed, and an electric foot shock (0.75 mA, 50 Hz, 3 s) was delivered via a constant-current stimulator [41]. The mouse was immediately removed and returned to its home cage. Both chambers were cleaned with 70% ethanol between trials to eliminate olfactory cues. Eight hours later, a retention trial was conducted under identical conditions, except that no foot shock was delivered. The latency to enter the dark chamber was recorded as an index of memory retention. The maximum latency was set at 300 s [42].

### Determination of Acetylcholine (ACh) Content and Acetylcholinesterase (AChE) Activity

The ACh content in hippocampal homogenates was quantified using a modified method of Vincent *et al*. [43]. Briefly, 20 μl of supernatant from brain homogenates and 50 μl of 1% hydroxylamine were added to each well of a 96-well plate. After 15 min of incubation at room temperature, 250 μl of FeCl_3_ solution (in 0.1 N HCl, pH 1.2 ± 0.2) was added. Physostigmine (1.5 mmol/l) was included to inhibit acetylcholinesterase (AChE) activity. After an additional 2 min incubation, absorbance was measured at 540 nm using a spectrophotometer, and values were calibrated against a blank. AChE activity was determined using a modified colorimetric method described by Ellman *et al*. [44]. Hippocampal tissue was homogenized (10%, w/v) in 30 mM sodium phosphate buffer (pH 7.0), followed by centrifugation at 10,000 ×*g* for 5 min at 4°C. For each sample, 10 μl of supernatant, 20 μl of 0.01 mM 5,5'-dithiobis(2-nitrobenzoic acid) (DTNB), and 0.1 mM sodium phosphate buffer (pH 8.0) were added to wells of a 96-well plate and incubated for 5 min at 26°C. The reaction was initiated by adding 10 μl of 0.1 M acetylthiocholine chloride. After 30 min, absorbance was read at 540 nm [45].

### Enzyme-Linked Immunosorbent Assay (ELISA) and TUNEL Staining

Cultured hippocampal neurons were incubated for 24 h, and the levels of TNF-α and IFN-β in the culture supernatant, as well as iNOS in the cell lysate, were quantified using commercially available ELISA kits, as previously described [46]. Briefly, 96-well plates pre-coated with anti-TNF-α, anti-IFN-β, or anti-iNOS antibodies were incubated with 100 μl of either supernatant or lysate per well for 1 h at 37°C. After incubation, the plates were washed three times with washing buffer and then incubated with streptavidin–HRP conjugate for 30 min at 37°C. Following another set of three washes, 3,3',5,5'-tetramethylbenzidine (TMB) substrate was added and allowed to react for 10 min at 37°C. The reaction was terminated with stop solution, and absorbance was measured at 450 nm using a microplate reader (Molecular Devices, USA). Terminal deoxynucleotidyl transferase dUTP nick end labeling (TUNEL) staining was performed using the In Situ Cell Death Detection Kit (Roche, Germany) according to the manufacturer’s instructions.

### RNA Preparation and Real-Time PCR Analysis

Total RNA was extracted from each sample using the easy-BLUE kit (iNtRON Biotech, Republic of Korea) according to the manufacturer’s protocol. RNA samples were treated with DNase I prior to cDNA synthesis and quantified by ultraviolet spectrophotometry. First-strand cDNA was synthesized from 2 μg of total RNA using gene-specific antisense primers and a first-strand synthesis mix (iNtRON Biotech) containing 20 mM Tris-HCl (pH 8.4), 50 mM KCl, 2.5 mM MgCl_2_, 10 mM dithiothreitol, 0.25 mM dNTPs, and 100 units of Moloney murine leukemia virus reverse transcriptase. Quantitative real-time PCR (qRT-PCR) was performed using the iCycler iQ real-time detection system (Bio-Rad Laboratories, USA) and iQ SYBR Green Supermix (Bio-Rad). The primer sequences used for the amplification of TLR4, NF-κB, and GAPDH are listed in Table 2. PCR reactions (25 μl) contained 12.5 μl SYBR Green Master Mix, 300 nM of each primer, 1 μl cDNA, and distilled water. The cycling protocol consisted of an initial denaturation at 95°C for 10 min, followed by 40 cycles of 95°C for 15 sec and 60°C for 60 sec. Melting curve analysis was performed to confirm the specificity of amplification. Relative mRNA expression levels were normalized to GAPDH. Ct values were analyzed using iQ5 optical system software (Bio-Rad). Primer efficiency was determined from standard curves (E = 10^(-1/slope)) based on serial dilutions of cDNA.

### Western Blot Analysis

Cultured hippocampal neurons were treated with various concentrations of CL-80 or ar-turmerone. After treatment, cells were washed twice with ice-cold phosphate-buffered saline (PBS, pH 7.4), collected, and lysed in SDS sample buffer (62.5 mM Tris-HCl, pH 6.8, 2% SDS, 10% glycerol, 5% 2-mercaptoethanol, 0.02% bromophenol blue). For *in vivo* samples, Aβ_1-42_-injected rats were sacrificed, and brains were rapidly dissected and stored at −70°C. Hippocampal tissues were homogenized in SDS sample buffer, centrifuged at 1,200 ×*g* for 10 min at 4°C, and the supernatants were collected. All protein samples were denatured by boiling at 100°C for 5 min. Equal amounts of protein were subjected to electrophoresis on 15% SDS–polyacrylamide gels and transferred onto polyvinylidene difluoride (PVDF) membranes (Hybond ECL, Amersham Pharmacia Biotech Inc., USA). Membranes were blocked in Tris-buffered saline containing 0.05% Tween-20 (TBST) and 5% bovine serum albumin (BSA) for 1 h at room temperature, then incubated overnight at 4°C with the following primary antibodies: anti-TLR4, anti- NF-κB, and anti-phospho-NF-κB (pNF-κB) (all at 1:1000 dilution). After washing in TBST, membranes were incubated for 1 h at room temperature with HRP-conjugated secondary antibodies (anti-rabbit or anti-mouse IgG, 1:10,000 dilution). Immunoreactive bands were visualized using the enhanced chemiluminescence (ECL) Western blot detection system (Pierce, USA).

### Statistical Analysis

Data are presented as means ± standard deviation (S.D.). Statistical significance was determined using one-way analysis of variance (ANOVA) or Student’s *t*-test, followed by Duncan’s multiple range post hoc test when appropriate. All statistical analyses were performed using GraphPad Prism (version 5.0, GraphPad Software, USA). *p*-values less than 0.05 were considered statistically significant.

## Results

### Extraction and Fractionation of *Curcuma longa* and Identification of ar-Turmerone

**Fig. 1:**
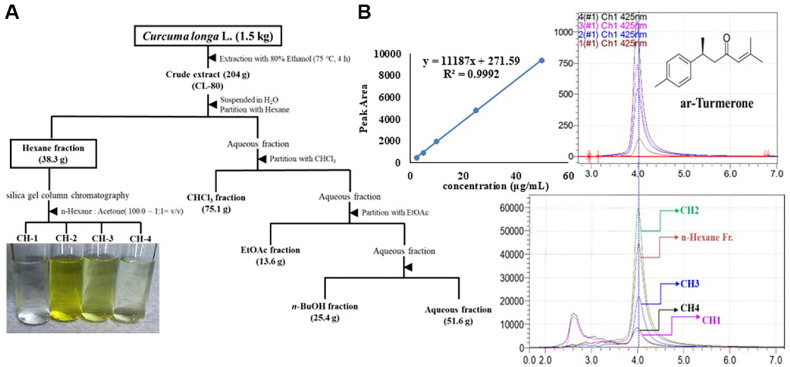
Extraction and chromatographic identification of ar-turmerone from *Curcuma longa*. (**A**) Schematic diagram of the extraction and fractionation procedure. Dried rhizomes of *Curcuma longa* were extracted with 80% ethanol to produce the CL-80 extract, followed by successive solvent partitioning using *n*-hexane, chloroform, ethyl acetate, and nbutanol. (**B**) High-performance liquid chromatography (HPLC) chromatograms of each subfraction (CH-1 to CH-4) from silica gel column chromatography of the *n*-hexane fraction. The major peak corresponding to ar-turmerone was identified in CH-2 based on UV absorbance at 425 nm and comparison with the authentic standard.

To identify the most bioactive extract of *C. longa*, five different extraction methods were tested: cold water extract (CL-CW), hot water extract (CL-HW), 20% ethanol extract (CL-20), 50% ethanol extract (CL-50), and 80% ethanol extract (CL-80). Among these, CL-80 exhibited the strongest biological activity, as demonstrated in the subsequent experimental results. The overall process for obtaining CL-80, including solvent partitioning and column chromatographic fractionation, is illustrated in Fig. 1A. Following 80% ethanol extraction, CL-80 was sequentially partitioned with *n*-hexane, chloroform, ethyl acetate, and *n*-butanol. Among these, the *n*-hexane fraction was found to contain the highest concentration of the active compound ar-turmerone, as determined by HPLC analysis (Fig. 1B). Further separation of the *n*-hexane fraction using silica gel column chromatography yielded four subfractions (CH-1 to CH-4). HPLC analysis of these subfractions revealed that CH-2 contained the greatest amount of ar-turmerone. The compound was identified based on its retention time and UV absorbance at 425 nm, in comparison with an authentic standard. The retention time of ar-turmerone was approximately 3.82 min. The analysis was completed within 7 min, and the full chromatograms are presented in Fig. 1B. Quantification using the external calibration curve (R² = 0.9992) revealed that ar-turmerone was present at 24.6 mg/g in CL-80, 2.6 mg/g in CH-1, 12.1 mg/g in the *n*-hexane fraction, and 17.0 mg/g in the CH-2 subfraction.

### Neuroprotective Effects of *C. longa* Extracts against Aβ-Induced Cytotoxicity

**Fig. 2:**
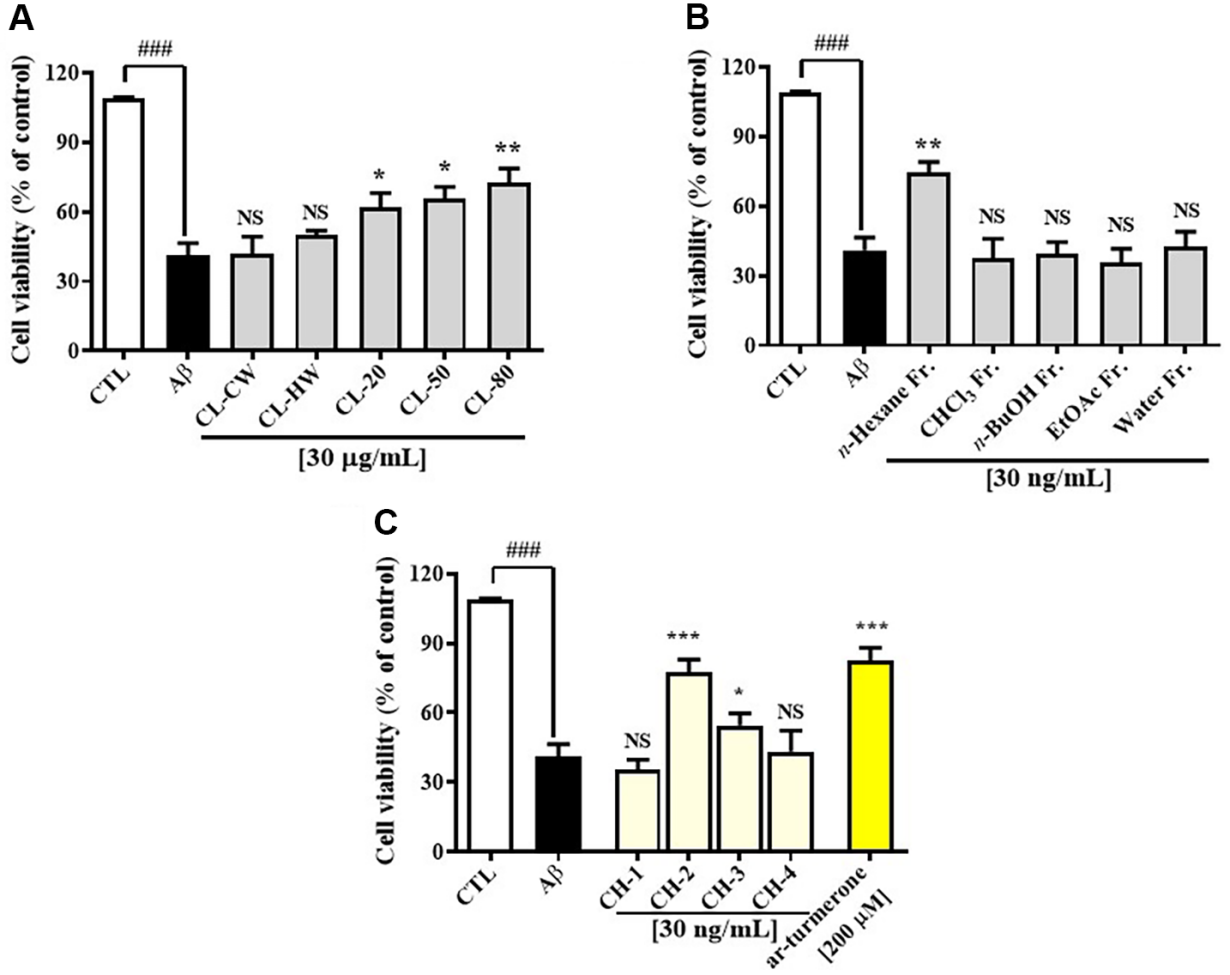
Screening of *Curcuma longa* extracts and fractions for neuroprotective effects against amyloid-β_25-35_-induced cytotoxicity. (**A**) Primary rat hippocampal neurons were treated with various *Curcuma longa* extracts prepared using cold water (CL-CW), hot water (CL-HW), 20% ethanol (CL-20), 50% ethanol (CL-50), and 80% ethanol (CL-80). Cell viability was measured after exposure to amyloid-β_25-35_ (50 μM) using a standard colorimetric assay. (**B**) Comparison of neuroprotective effects among solvent-partitioned fractions (*n*-hexane, chloroform, ethyl acetate, *n*-butanol) derived from the 80% ethanol extract (CL-80). (**C**) Subfractions (CH-1 to CH-4) of the *n*-hexane extract were obtained by silica gel chromatography and evaluated for protective effects against amyloid-β-induced toxicity. All experiments were independently performed using three separate culture plates, and each measurement was repeated in technical triplicate. Data are presented as mean ± standard deviation (S.D.). ***p < 0.001, **p < 0.01, *p < 0.05 vs. amyloid-β group; ^###^*p* < 0.001 vs. untreated control group; NS: not significant.

To evaluate the neuroprotective potential of various *C. longa* extracts, primary cultured rat hippocampal neurons were exposed to Aβ-induced toxicity and subsequently treated with extracts obtained under different extraction conditions: CL-CW, CL-HW, CL-20, CL-50, and CL-80, each at a final concentration of 30 μg/ml. As shown in Fig. 2A, Aβ_(25-35)_ exposure significantly reduced cell viability to 40.08 ± 2.67% (*p* < 0.001). Among the tested extracts, CL-CW and CL-HW did not confer significant protection, while CL-20 and CL-50 moderately improved viability to 62.06 ± 3.02% and 66.74 ± 2.53%, respectively (*p* < 0.05). Notably, CL-80 exhibited the most potent neuroprotective effect, restoring cell viability to 72.69 ± 2.97% (*p* < 0.01). Based on this superior activity, CL-80 was subjected to solvent partitioning using *n*-hexane, chloroform, ethyl acetate, and *n*-butanol. Among these, only the *n*-hexane fraction significantly attenuated Aβ-induced cytotoxicity (74.46 ± 2.28%, *p* < 0.01), whereas the other fractions had no significant effect (*p* > 0.05) (Fig. 2B). The *n*-hexane fraction was further separated by silica gel column chromatography into four subfractions (CH-1 to CH-4). As illustrated in Fig. 2C, CH-3 moderately improved cell viability (54.56 ± 2.60%, *p* < 0.05), while CH-1 and CH-4 showed no significant effects. CH-2 displayed the most pronounced neuroprotective effect, increasing viability to 77.69 ± 2.63% (*p* < 0.001). HPLC analysis confirmed that CH-2 contained the ar-turmerone, as identified by retention time (Fig. 1B). These results strongly suggest that ar-turmerone is the principal bioactive compound in *C. longa* responsible for the observed neuroprotection against Aβ-induced neuronal damage.

### CL-80 and ar-Turmerone Protect Against Aβ-Induced Neurotoxicity and Attenuate Inflammatory Cytokine Production in Cultured Hippocampal Neurons

**Fig. 3:**
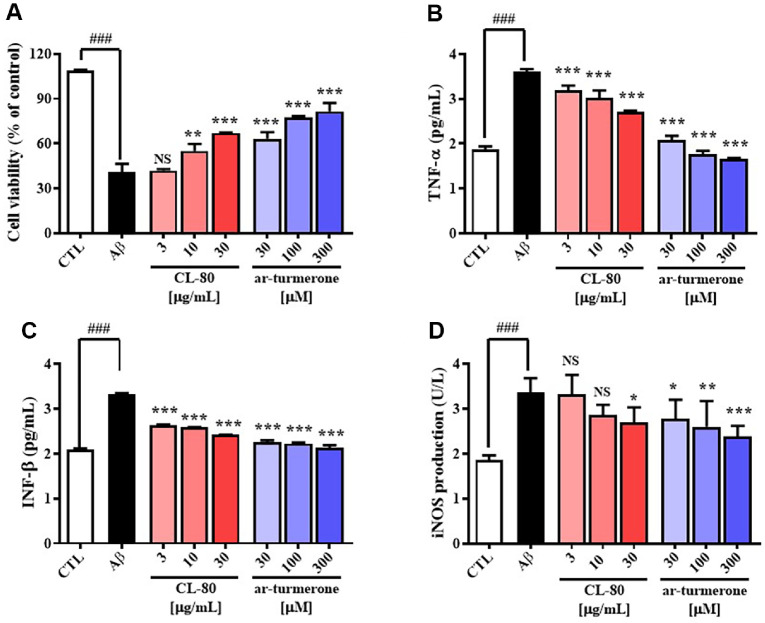
Effects of CL-80 and ar-turmerone on amyloid-β_25-35_-induced cytotoxicity and inflammatory cytokine expression in cultured hippocampal neurons. (**A**) Cell viability was measured following treatment with the 80% ethanol extract of *Curcuma longa* (CL-80; 3, 10, 30 μg/ml) or ar-turmerone (30, 100, 300 μM) in the presence of amyloid- β_25-35_. (**B–D**) Levels of tumor necrosis factor-alpha (TNF-α), interferon-beta (IFN-β), and inducible nitric oxide synthase (iNOS) were measured in the culture supernatant or lysates using enzyme-linked immunosorbent assays (ELISA). All experiments were performed using three independent culture plates, with each assay repeated in triplicate. Data are shown as mean ± S.D. ***p < 0.001, **p < 0.01, *p < 0.05 vs. amyloid-β group; ^###^*p* < 0.001 vs. untreated control group; NS: not significant.

To verify the neuroprotective effects of CL-80 and its major active constituent, ar-turmerone, which were identified in a prior screening (Fig. 2), primary hippocampal neurons were treated with various concentrations of CL-80 or ar-turmerone in the presence of Aβ_(25-35)_ (50 μM). As shown in Fig. 3A, CL-80 significantly attenuated Aβ-induced cytotoxicity in a concentration-dependent manner. At concentrations of 10 and 30 μg/ml, CL-80 increased cell viability from 40.08 ± 2.67% (Aβ group) to 55.20 ± 2.28% (*p* < 0.01) and 67.56 ± 0.16% (*p* < 0.001), respectively. Similarly, ar-turmerone treatment at 30, 100, and 300 μM enhanced viability to 63.24 ± 2.14%, 77.70 ± 0.37%, and 81.57 ± 2.78%, respectively (all *p* < 0.001), indicating strong neuroprotection across all doses tested. Based on these protective concentrations, inflammatory cytokines were next assessed. Aβ treatment significantly elevated TNF-α levels in the culture supernatant to 3.61 ± 0.02 pg/ml. CL-80 treatment at 3, 10, and 30 μg/ml significantly decreased TNF-α to 3.19 ± 0.06 (*p* < 0.01), 3.02 ± 0.20 (*p* < 0.01), and 2.71 ± 0.09 pg/ml (*p* < 0.001), respectively. Ar-turmerone also significantly reduced TNF-α at 30, 100, and 300 μM to 2.07 ± 0.02, 1.77 ± 0.05, and 1.67 ± 0.04 pg/ml, respectively (all *p* < 0.001) (Fig. 3B). A similar trend was observed for IFN-β, which increased to 3.33 ± 0.01 pg/ml upon Aβ stimulation. CL-80 reduced IFN-β levels in a dose-dependent manner: 2.63 ± 0.01 pg/ml at 3 μg/ml, 2.58 ± 0.01 pg/ml at 10 μg/ml, and 2.42 ± 0.01 pg/ml at 30 μg/ml (all *p* < 0.001). Ar-turmerone treatments at 30, 100, and 300 μM resulted in significant suppression of IFN-β to 2.26 ± 0.02, 2.23 ± 0.01, and 2.14 ± 0.03 pg/ml, respectively (all *p* < 0.001) (Fig. 3C). Moreover, iNOS activity in the cell lysate, which was elevated to 3.36 ± 0.13 U/l by Aβ, was significantly reduced by CL-80 at 30 μg/ml to 2.70 ± 0.14 U/l (*p* < 0.05), and by ar-turmerone at 30, 100 and 300 ng/ml to 2.78 ± 0.18 U/l (*p* < 0.05), 2.60 ± 0.23 U/l (*p* < 0.01) and 2.38 ± 0.10 U/l (*p* < 0.001), respectively (Fig. 3D). Collectively, these results confirm that both CL-80 and ar-turmerone exert potent neuroprotective effects against Aβ-induced cytotoxicity and significantly attenuate inflammatory responses by suppressing TNF-α, IFN-β, and iNOS expression in a concentration-dependent manner.

### CL-80 and ar-Turmerone Suppress Aβ-Induced TLR4 and NF-κB mRNA and Protein Expression in Cultured Hippocampal Neurons

**Fig. 4:**
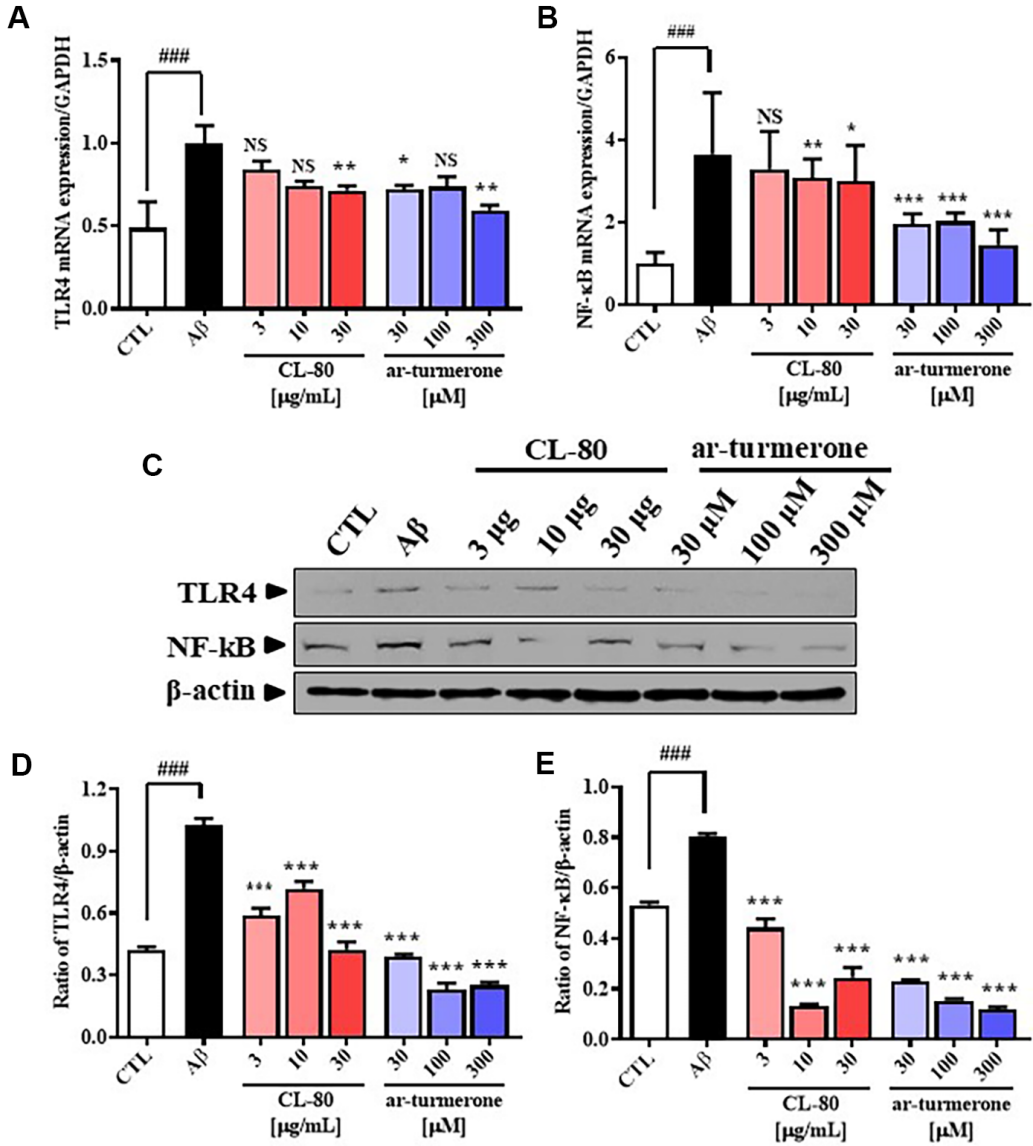
CL-80 and ar-turmerone suppress Toll-like receptor 4 and NF-κB expression in amyloid-β_25-35_- stimulated hippocampal neurons. (**A–B**) mRNA expression levels of Toll-like receptor 4 (TLR4) and nuclear factor kappa B (NF-κB) were measured by quantitative reverse transcription polymerase chain reaction (qRT-PCR). (**C**) Representative Western blot images of TLR4, NF-κB, and β-actin proteins from cell lysates. (**D–E**) Densitometric analysis of protein expression was conducted to quantify relative levels of TLR4 and NF-κB following treatment with CL-80 or ar-turmerone. Each experiment was independently conducted using three separate culture plates, and each sample was analyzed in triplicate. Data are expressed as mean ± S.D. ***p < 0.001, **p < 0.01, *p < 0.05 vs. amyloid-β group; ^###^*p* < 0.001 vs. untreated control group.

To explore whether CL-80 and ar-turmerone regulate Aβ-induced neuroinflammation through the TLR4/NF- κB pathway, mRNA and protein expression levels of TLR4 and NF-κB were measured in primary cultured hippocampal neurons. As shown in Fig. 4A and 4B, treatment with Aβ_(25-35)_ (50 μM) significantly increased the mRNA levels of TLR4 and NF-κB compared to untreated controls. CL-80 treatment at 30 μg/ml significantly reduced TLR4 mRNA expression to 0.71 ± 0.02 (*p* < 0.01), while ar-turmerone treatment at 30 and 300 μM resulted in reductions to 0.71 ± 0.02 (*p* < 0.05) and 0.59 ± 0.03 (*p* < 0.01), respectively (Fig. 4A). Additionally, NF-κB mRNA levels were significantly decreased by CL-80 at 10 and 30 μg/ml (3.07 ± 0.45 and 2.99 ± 0.87, *p* < 0.01 and *p* < 0.05, respectively), and by ar-turmerone at 100 μM (2.02 ± 0.20, *p* < 0.001) (Fig. 4B).

At the protein level, Aβ markedly increased the expression of TLR4 and NF-κB, which was effectively suppressed by both CL-80 and ar-turmerone treatments. As shown in Fig. 4C, TLR4 protein levels were significantly downregulated by CL-80 at 3, 10, and 30 μg/ml (0.59 ± 0.02, 0.72 ± 0.02, and 0.43 ± 0.02, respectively; all *p* < 0.001), and by ar-turmerone at 30, 100, and 300 μM (0.39 ± 0.01, 0.23 ± 0.01, and 0.25 ± 0.01; all *p* < 0.001). Similarly, NF- κB protein expression was significantly inhibited by CL-80 (3 μg/ml: 0.44 ± 0.02; 10 μg/ml: 0.13 ± 0.01; 30 μg/ml: 0.25 ± 0.02; all *p* < 0.001) and ar-turmerone (30 μM: 0.23 ± 0.01; 100 μM: 0.16 ± 0.001; 300 μM: 0.12 ± 0.01; all *p* < 0.001) (Fig. 4D). These findings suggest that CL-80 and ar-turmerone effectively downregulate both transcriptional and translational expression of TLR4 and NF-κB, thereby attenuating Aβ-induced activation of the TLR4/NF-κB signaling pathway in hippocampal neurons.

### CL-80 and ar-Turmerone Ameliorate Aβ_1-42_-Induced Memory Impairment in the Morris Water Maze Test

**Fig. 5:**
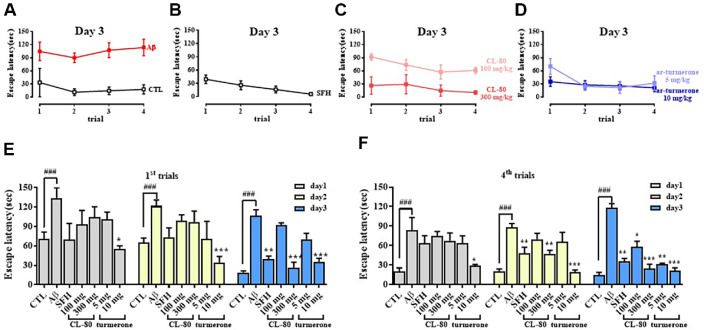
Effects of CL-80 and ar-turmerone on spatial learning in amyloid-β_1-42_-injected mice using the Morris water maze test. (**A**) Escape latency across four trials on day 3 after hippocampal injection of amyloid-β_1-42_ (4 μg/mouse). (**B**) Positive control group treated with silk fibroin hydrolysate (SFH, 100 mg/kg). (**C–D**) Escape latency in groups treated with CL- 80 (100 or 300 mg/kg) or ar-turmerone (5 or 10 mg/kg). (**E–F**) Average escape latency during the first (**E**) and fourth (**F**) trials in the acquisition phase. Behavioral tests were performed with 7 mice per group. Data are presented as mean ± S.D. ***p < 0.001, **p < 0.01, *p < 0.05 vs. amyloid-β group; ^###^*p* < 0.001 vs. untreated control group.

To evaluate the effects of CL-80 and ar-turmerone on spatial learning and memory deficits induced by Aβ_1-42_, we conducted the Morris water maze test over four trials per day for 3 days (Fig. 5A–5D). The Aβ_1-42_-injected group exhibited prolonged escape latencies throughout the trials, indicating impaired spatial learning compared to the control group. On the third day, both CL-80 and ar-turmerone treatment groups showed significantly improved performance. During the first trial (Fig. 5E), the escape latency of the Aβ group was 106.55 ± 22.57 s (*p* < 0.001). In contrast, treatment with CL-80 at 300 mg/kg significantly reduced the latency to 26.60 ± 19.93 s (*p* < 0.001). Similarly, ar-turmerone at 10 mg/kg also shortened the escape time to 35.45 ± 10.91 s (*p* < 0.001).

In the final trial on day 3 (Fig. 5F), escape latencies further decreased in the treated groups. The Aβ group maintained a high latency of 117.94 ± 16.40 s, whereas the CL-80 100 mg/kg and 300 mg/kg groups showed significant reductions to 57.85 ± 15.06 s (*p* < 0.05) and 24.95 ± 10.93 s (*p* < 0.001), respectively. Likewise, ar-turmerone at 5 mg/kg and 10 mg/kg decreased latencies to 31.39 ± 3.43 s (*p* < 0.01) and 21.26 ± 8.98 s (*p* < 0.001), respectively.

**Fig. 6:**
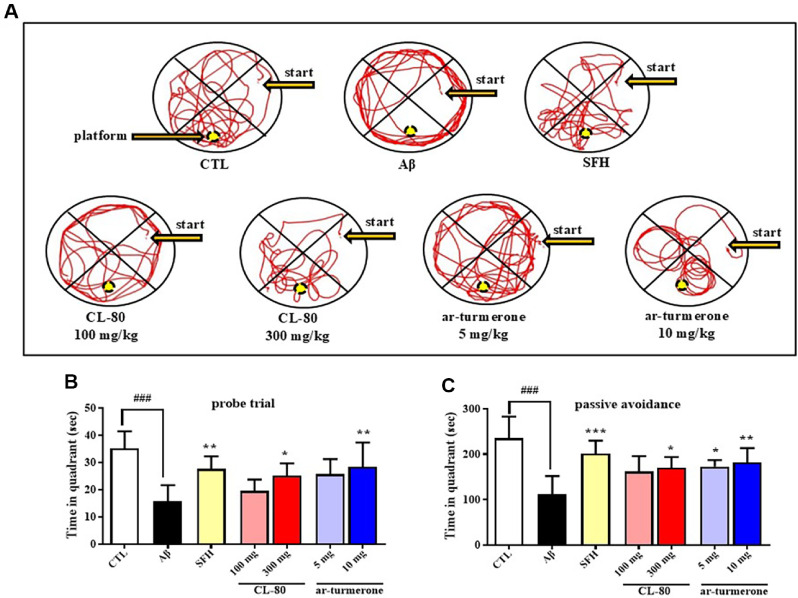
CL-80 and ar-turmerone improve spatial memory and passive avoidance behavior in amyloid-β_1-42_-injected mice. (**A**) Representative swimming traces from the 90-second probe trial on day 4 of the Morris water maze test. Time spent in the target quadrant was used as a measure of memory retention. (**B**) Quantification of time spent in the target quadrant. (**C**) Passive avoidance test was conducted to evaluate contextual memory. Latency to enter the dark compartment was measured 8 h after the acquisition trial. Each group consisted of 7 mice. Data are shown as mean ± S.D. ***p < 0.001, **p < 0.01, *p < 0.05 vs. amyloid-β group; ^###^*p* < 0.001 vs. untreated control group.

To evaluate spatial memory retention, the Morris water maze probe trial was conducted on day 4 after the acquisition phase. Representative swim traces from each group are shown in Fig. 6A. The Aβ_1-42_-injected group exhibited a notable impairment in spatial memory, as evidenced by a significant reduction in the time spent in the target quadrant (15.65 ± 6.06 s, *p* < 0.001) compared to the control group (35.18 ± 6.31 s). In contrast, treatment with CL-80 or ar-turmerone ameliorated this impairment. Specifically, the CL-80 300 mg/kg groups spent 25.11 ± 4.56 s (*p* < 0.05) in the target quadrant. Similarly, ar-turmerone-treated mice showed a significant improvement, spending 25.71 ± 5.50 s (5 mg/kg, *p* < 0.05) and 28.33 ± 3.99 s (10 mg/kg, *p* < 0.01) in the target area (Fig. 6B). Based on HPLC standardization, 300 mg/kg of CL-80 corresponds to ~7.4 mg/kg of ar-turmerone, allowing an approximate comparison with the ar-turmerone group. These findings suggest that both CL-80 and ar-turmerone effectively attenuate Aβ-induced spatial memory deficits in a dose-dependent manner, supporting their neuroprotective and cognition-enhancing properties.

### CL-80 and ar-Turmerone Attenuate Aβ_1-42_-Induced Cognitive Impairments in the Passive Avoidance Test

To further investigate the cognitive-protective effects of CL-80 and ar-turmerone, a passive avoidance test was conducted (Fig. 6C). The Aβ_1-42_-injected group exhibited a significantly shortened latency to enter the dark compartment (111.81 ± 40.19 s, *p* < 0.001) compared to the control group (235.67 ± 47.57 s), indicating impaired memory retention. However, treatment with CL-80 or ar-turmerone significantly ameliorated the Aβ_1-42_-induced cognitive deficit. Specifically, latency times were increased in mice treated with 100 mg/kg CL-80 (161.72 ± 34.14 s, *p* < 0.01) and 300 mg/kg CL-80 (170.21 ± 23.86 s, *p* < 0.01). Similarly, ar-turmerone administration at 5 mg/kg (172.90 ± 14.46 s, *p* < 0.05) and 10 mg/kg (181.52 ± 32.16 s, *p* < 0.01) significantly improved memory performance. Notably, the positive control group treated with SFH showed a robust protective effect (201.13 ± 28.96 s, *p* < 0.001). These findings demonstrate that both CL-80 and ar-turmerone effectively alleviate Aβ_1-42_- induced deficits in learning and memory.

### Effect of CL-80 and ar-Turmerone on AChE Activity and ACh Content in Aβ_1-42_-Injected Mice

**Fig. 7:**
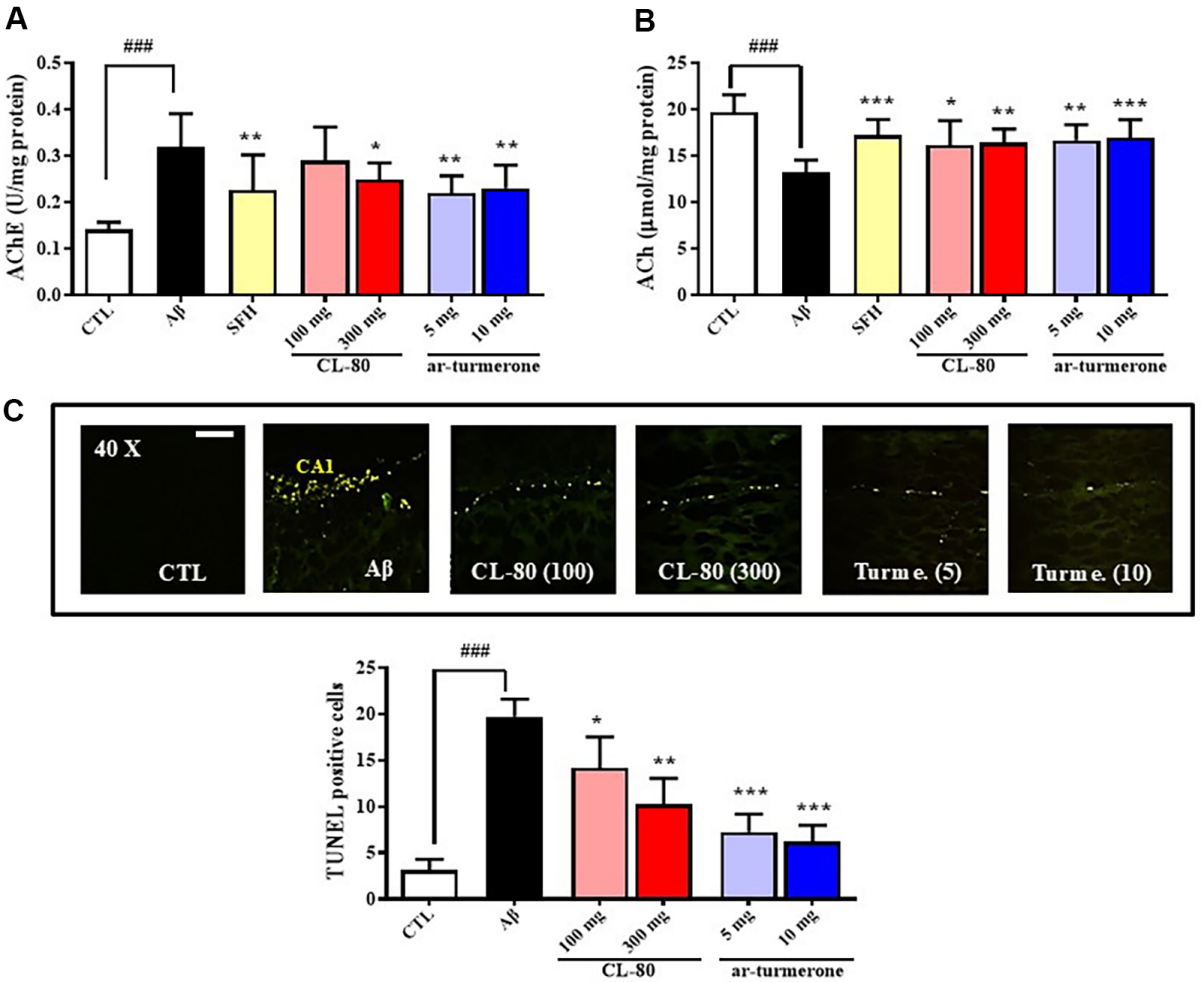
Effects of CL-80 and ar-turmerone on hippocampal acetylcholine levels, acetylcholinesterase activity, and neuronal apoptosis in amyloid-β_1-42_-injected mice. (**A**) Acetylcholinesterase (AChE) activity was assessed in the same samples using a modified Ellman’s assay. (**B**) Acetylcholine (ACh) levels were measured in hippocampal homogenates using the hydroxylamine–ferric chloride colorimetric method. (**C**) Representative hippocampal sections were stained using the terminal deoxynucleotidyl transferase dUTP nick end labeling (TUNEL) method to detect apoptotic nuclei. Fluorescence microscopy was performed at 40× magnification. TUNEL-positive cells were quantified in randomly selected fields to assess neuronal apoptosis. Each group included 7 mice. Data are expressed as mean ± S.D. ***p < 0.001, **p < 0.01, *p < 0.05 vs. amyloid- β group; ^###^*p* < 0.001 vs. untreated control group. Scale bar = 50 μm.

To assess the impact of CL-80 and ar-turmerone on cholinergic dysfunction induced by Aβ_1-42_, we measured AChE activity and ACh content in hippocampal tissues. Aβ_1-42_ injection significantly increased AChE activity to 0.32 ± 0.07 U/mg protein (*p* < 0.001), approximately 2.3-fold higher than the control group (0.14 ± 0.02 U/mg protein, Fig. 7A). Conversely, the ACh level was significantly decreased in the Aβ-treated group (13.22 ±1.33 μmol/mg protein) compared to control (19.71 ± 1.88 μmol/mg protein, *p* < 0.001, Fig. 7B). Administration of CL-80 at 100 and 300 mg/kg effectively attenuated the AChE activity to 0.29 ± 0.07 and 0.25 ± 0.04 U/mg protein, respectively, with statistical significance at 300 mg/kg (*p* < 0.05). Similarly, ar-turmerone at 5 and 10 mg/ kg significantly reduced AChE activity to 0.22 ± 0.04 and 0.23 ± 0.05 U/mg protein (*p* < 0.01). In parallel, ACh levels were significantly restored following both treatments. In the CL-80 100 and 300 mg/kg groups, ACh levels were elevated to 16.15 ± 2.65 (*p* < 0.05) and 16.44 ± 1.47 μmol/mg protein (*p* < 0.01), respectively. Likewise, arturmerone at 5 and 10 mg/kg significantly increased ACh content to 16.97 ± 1.71 (*p* < 0.01) and 16.97 ± 1.94 μmol/mg protein (*p* < 0.001). Collectively, these data demonstrate that both CL-80 and ar-turmerone effectively counteract Aβ_1-42_-induced cholinergic impairment by reducing AChE hyperactivity and restoring synaptic ACh levels, supporting their potential neuroprotective efficacy in AD models.

### TUNEL Assay Reveals Anti-Apoptotic Effects of CL-80 and Ar-Turmerone in Aβ_1−42_-Injected Mice

To assess neuronal apoptosis in the CA1 region of hippocampal tissue, TUNEL staining was performed following Aβ_1-42_ injection. As shown in Fig. 7C, the number of TUNEL-positive cells was significantly increased in the Aβ group (19.71 ± 6.60 cells), compared to the control group (3.01 ± 0.42 cells; *p* < 0.001). This result indicates a robust induction of neuronal apoptosis by Aβ_1-42_. However, treatment with CL-80 markedly attenuated this effect in a dose-dependent manner. The number of TUNEL-positive cells was significantly reduced to 14.15 ± 1.07 cells (*p* < 0.05) and 10.23 ± 0.89 cells (*p* < 0.01) in the CL-80 100 and 300 mg/kg groups, respectively. Similarly, arturmerone treatment at doses of 5 and 10 mg/kg significantly decreased TUNEL-positive cells to 7.27 ± 0.61 and 6.17 ± 0.57, respectively (both *p* < 0.001). These findings suggest that both CL-80 and ar-turmerone exert strong anti-apoptotic effects in Aβ-induced neuronal injury.

### Effects of CL-80 and ar-Turmerone on TLR4 and NF-κB mRNA and Protein Levels in the Hippocampus

**Fig. 8:**
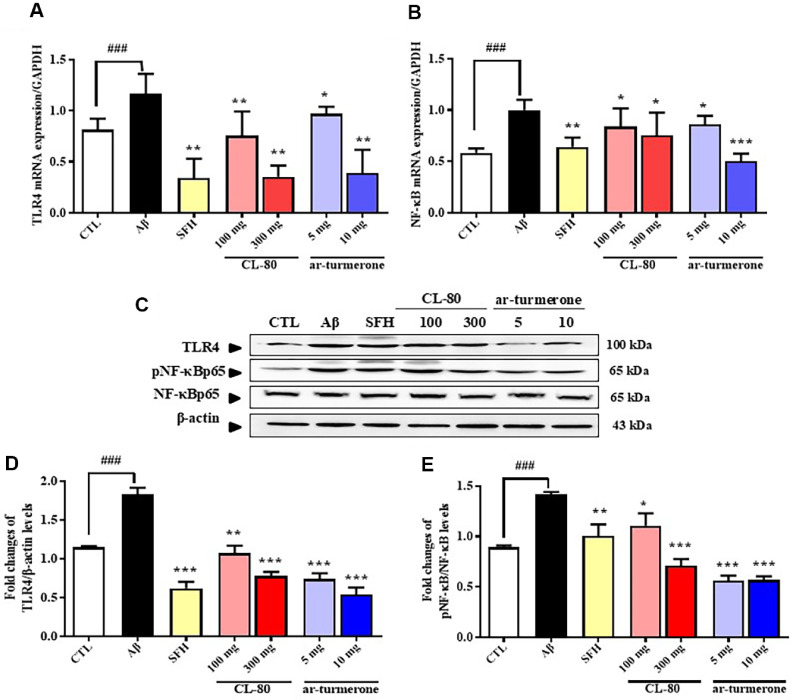
Effects of CL-80 and ar-turmerone on mRNA expression and protein levels of Toll-like receptor 4, NF-κB, and phosphorylated NF-κB in hippocampal tissue of amyloid-β_1-42_-injected mice. (**A–B**) Quantitative realtime polymerase chain reaction (qRT-PCR) was used to assess the mRNA levels of Toll-like receptor 4 (TLR4) (**A**) and nuclear factor kappa B (NF-κB) (**B**) in hippocampal tissue. Expression levels were normalized to glyceraldehyde 3-phosphate dehydrogenase (GAPDH) mRNA. (**C**) Western blot images showing protein levels of TLR4, NF-κB, and phosphorylated NF-κB (pNF-κB). (**D**) Densitometric quantification of TLR4 protein levels normalized to β-actin. (**E**) Densitometric quantification of phosphorylated NF-κB in nuclear fractions, indicating transcriptional activation. Each group included 7 mice. Data are expressed as mean ± S.D. ***p < 0.001, **p < 0.01, *p < 0.05 vs. amyloid-β group; ^###^*p* < 0.001 vs. untreated control group.

To investigate whether CL-80 and ar-turmerone regulate the TLR4/NF-κB signaling pathway *in vivo*, we measured mRNA and protein levels of TLR4 and NF-κB in the hippocampus of Aβ_1-42_-injected mice. As shown in Fig. 8A and 8B, Aβ_1-42_ significantly increased the mRNA expression of both TLR4 (1.17 ± 0.19, *p* < 0.001) and NF-κB (1.00 ± 0.10, *p* < 0.001) compared to the control group (TLR4: 0.82 ± 0.11; NF-κB: 0.58 ± 0.04). Treatment with CL- 80 (100 and 300 mg/kg) or ar-turmerone (5 and 10 mg/kg) markedly reduced TLR4 mRNA expression in a dosedependent manner (Fig. 8A). Treatment with CL-80 (100 and 300 mg/kg) or ar-turmerone (5 and 10 mg/kg) also markedly reduced NF-κB mRNA expression (Fig. 8B).

At the protein level, the expression of TLR4 was elevated following Aβ injection (1.16 ± 0.11), and significantly reduced by CL-80 and ar-turmerone administration (Fig. 8C and 8D). Interestingly, while NF-κB mRNA levels were also elevated by Aβ, they were not significantly suppressed by either compound, and in some cases appeared to increase slightly, suggesting that CL-80 and ar-turmerone do not inhibit NF-κB at the cytoplasmic protein levels (Fig. 8C). However, analysis of phosphorylated NF-κB (p-NF-κB) in nuclear fractions revealed a marked activation of NF-κB following Aβ injection (1.42 ± 0.23, *p* < 0.001) (Fig. 8E). This nuclear activation was significantly attenuated by CL-80 (100 mg/kg: 1.11 ± 0.12, *p* < 0.05; 300 mg/kg: 0.71 ± 0.07, *p* < 0.001) and ar-turmerone (5 mg/kg: 0.56 ± 0.05; 10 mg/kg: 0.57 ± 0.04; both *p* < 0.001) (Fig. 8D). Taken together, these results suggest that although Aβ_1-42_ enhances both transcription and nuclear activation of NF-κB, CL-80 and ar-turmerone primarily inhibit inflammatory signaling by suppressing NF-κB phosphorylation and its nuclear translocation, rather than altering cytoplasmic protein levels. This finding is consistent with our *in vitro* data (Fig. 3), where treatment with CL-80 or ar-turmerone significantly downregulated NF-κB target genes such as TNF-α, IFN-β, and iNOS. Therefore, the observed anti-inflammatory effects of CL-80 and ar-turmerone in the Aβ-induced model appear to be mediated via post-translational regulation of NF-κB activity, specifically through inhibition of its nuclear activation. While this interpretation aligns with the well-established NF-κB signaling mechanism, further studies are needed to identify the precise upstream kinases or regulatory proteins, such as IκB kinase (IKK) or MAPKs, that may be modulated by CL-80 and ar-turmerone to suppress NF-κB phosphorylation.

## Discussion

AD is a progressive neurodegenerative disorder characterized by memory loss, cognitive impairment, and neuronal degeneration. A central pathological hallmark of AD is the accumulation of Aβ peptides in the brain, which activate glial cells and initiate a cascade of neuroinflammatory responses that contribute to neuronal injury [2, 3]. Numerous studies have shown that AD progression and pathogenesis are closely associated with inflammation involving astrocytes [47] and microglia [48], both of which express TLRs [49] and contribute to the chronic neuroinflammatory environment characteristic of AD. Increasing evidence implicates innate immune receptors such as TLR4 and its downstream effector NF-κB as key mediators of this inflammatory process [11, 50]. Chronic inflammation mediated by glial cells is now widely recognized as a crucial driver of AD pathogenesis [7, 48]. These glial cells express pattern recognition receptors such as TLR4, which recognize Aβ aggregates and trigger pro-inflammatory gene expression via NF-κB activation. This, in turn, promotes the release of cytokines such as TNF-α, IL-1β, IFN-β, and the enzyme iNOS, ultimately leading to neurodegeneration [15, 51].

*C. longa* and its principal components, including curcumin and ar-turmerone, have long been studied for their anti-inflammatory and neuroprotective properties [24, 52]. Curcumin, the major component of *C. longa*, has been used in traditional medicine for centuries and has been proposed as a therapeutic agent for enhancing the clearance of toxic Aβ aggregates [52, 53]. It has demonstrated antioxidant and anti-inflammatory activities that protect against Aβ-induced toxicity and reduce microglial activation [26, 54-57]. While curcumin has been shown to attenuate Aβ-induced neurotoxicity and inhibit microglial activation, the bioactive sesquiterpenoid arturmerone has only recently emerged as a potential neuroprotective agent with favorable pharmacokinetic properties, including BBB permeability. Ar-turmerone is a sesquiterpenoid with favorable physicochemical properties, including moderate lipophilicity and low molecular weight, which support its ability to penetrate the BBB. Previous studies have confirmed its CNS bioavailability and functional activity *in vivo*. For instance, Zhou *et al*. and Hucklenbroich *et al*., demonstrated that ar-turmerone crosses the BBB and accumulates in brain tissue where it promotes neural stem cell proliferation [32, 36]. Likewise, Orellana-Paucar *et al*., observed significant CNS effects of ar-turmerone in both zebrafish and murine seizure models [35]. Based on these findings and the behavioral and biochemical outcomes observed in our Aβ_1-42_-injected mouse model, it is reasonable to infer that ar-turmerone reaches the brain in pharmacologically relevant concentrations. Future studies including direct quantification of brain tissue levels and pharmacokinetic profiling are warranted to further substantiate these observations.

In this study, both CL-80 and ar-turmerone demonstrated protective effects against Aβ_1-42_-induced neurotoxicity, with comparable trends observed across behavioral and molecular outcomes. Based on our HPLC standardization, the ar-turmerone content in CL-80 was determined to be 24.6 mg/g of dried extract (2.46 w/w%). Thus, oral administration of CL-80 at 300 mg/kg corresponds to an estimated ar-turmerone exposure of approximately 7.4 mg/kg. This value is within the same order of magnitude as the doses of ar-turmerone applied in our experiments, providing a rationale for the side-by-side comparison presented in Fig. 6. However, we acknowledge that this equivalence remains approximate because CL-80 is a complex phytochemical mixture containing multiple bioactive constituents in addition to ar-turmerone, such as curcumin and other volatile sesquiterpenes, which may act synergistically or independently. Therefore, the direct quantitative comparison between CL-80 and ar-turmerone should be interpreted with caution. While the estimated ar-turmerone equivalence supports the translational relevance of our findings, future studies are warranted to delineate the contribution of other phytoconstituents and to establish more precise dose–response relationships.

Furthermore, curcumin has been extensively studied for its interactions with TLRs/NF-κB signaling in various pathological conditions including inflammation [58], acute pancreatitis [59], and cerebral ischemia [60], the mechanistic roles of ar-turmerone in modulating this pathway, particularly in the context of AD, remain poorly understood. Therefore, this study aimed to explore the therapeutic potential of *C. longa*-derived CL-80 and arturmerone against Aβ-induced neuroinflammation and cognitive deficits by targeting the TLR4/NF-κB signaling pathway.

In this study, we demonstrated that CL-80, an 80% ethanolic extract of *C. longa*, and ar-turmerone significantly attenuated Aβ-induced neuroinflammatory responses both *in vitro* and *in vivo*. In primary cultured rat hippocampal neurons stimulated with Aβ_(25-35)_, both compounds dose-dependently suppressed the production of TNF-α, IFN- β, and iNOS. Neuroinflammation is primarily mediated by activated microglial cells responding to Aβ. These cells release a wide spectrum of pro-inflammatory mediators, including TNF-α, interleukin-1β (IL-1β), cyclooxygenase- 2 (COX-2), and iNOS, contributing to neuronal injury [61, 62]. However, the underlying signaling mechanisms that drive this response are not fully understood. While cytokines and adhesion molecules act as downstream effectors, pattern recognition receptors such as TLRs and transcription factors like NF-κB function as upstream regulators of the inflammatory cascade [15, 50]. Moreover, emerging evidence suggests that neurons themselves may directly contribute to neuroinflammation via intrinsic TLR/NF-κB signaling. To investigate these neuronintrinsic mechanisms, we utilized purely cultured primary hippocampal neurons and confirmed that CL-80 and ar-turmerone suppress Aβ-induced inflammatory signaling in neurons independent of glial influence.

Furthermore, treatment with CL-80 and ar-turmerone suppressed Aβ-induced upregulation of TLR4 and NF-κB at mRNA levels. These effects were replicated in Aβ_1-42_-injected mouse hippocampi, confirming that these agents modulate the TLR4/NF-κB axis at the transcriptional and post-transcriptional levels. TLRs are important surface receptors in innate immunity. Many studies have shown that AD pathogenesis and progression are associated with inflammation involving microglia and astrocytes, which express TLRs. Indeed, recent work has implicated TLR4 in neurodegeneration and disease progression in AD patients [62]. These receptors mediate activation of transcription factors such as NF-κB, interferon regulatory transcription factor 3 (IRF3), IRF5 and IRF7 and subsequent production of inflammatory cytokines, including interferon-β [12]. NF-κB is one of the central downstream transcription factors in TLR signaling pathways [12]. Accompanied by a significant increase of proinflammatory cytokines, NF-κB is activated in glial cells in patients with AD and these activated NF-κB also are found in neurons in areas surrounding Aβ plaque [51]. We found that the expression of TLR4 in both mRNA and protein levels were upregulated after Aβ treatment, but, CL-80 and ar-turmerone treatment reversed this trend. Consistent with previous studies [63, 64], we observed that ar-turmerone exert anti-inflammatory effects through NF-κB pathway inhibition, while our findings extend this by showing that ar-turmerone also suppresses the phosphorylation and nuclear translocation of NF-κB, a critical post-translational step in the inflammatory cascade [51]. Importantly, phosphorylated NF-κB in the nuclear fraction, reflecting its activated state, was also significantly suppressed by both treatments, despite cytoplasmic NF-κB protein levels remaining unchanged. These results suggest that CL-80 and ar-turmerone regulate NF-κB activity predominantly through modulation of its activation and nuclear localization rather than its total expression. This mechanism is consistent with previous studies in models of ischemia and inflammation [59, 60], supporting the hypothesis that ar-turmerone acts as a neuroimmune modulator in AD. Although Aβ_1-42_ injection markedly enhanced nuclear phosphorylation of NF- κB, as shown in Fig. 8E, this effect was significantly attenuated by both CL-80 and ar-turmerone, indicating suppression of NF-κB activation at the post-translational level. This observation implies potential upstream interference at the level of NF-κB regulatory kinases. Indeed, prior studies have reported that ar-turmerone modulates JNK and p38 MAPK signaling in LPS-stimulated BV-2 microglial cells [37], and that curcumin, a major component of CL-80, inhibits IκBα degradation and suppresses p38/JNK activation in Aβ-challenged neurons [29]. Based on these findings, it is plausible that the observed inhibition of p-NF-κB nuclear translocation in our study may involve upstream regulation of the IKK complex and/or MAPKs. However, it should be clearly acknowledged that the present work did not directly examine upstream intermediates such as phospho-IKKα/β, IκBα degradation, or MAPK activation (p38, JNK, ERK). This constitutes a limitation of our mechanistic interpretation, and thus our conclusion regarding TLR4/NF-κB signaling should be interpreted with caution. Future studies will specifically investigate whether CL-80 and ar-turmerone attenuate NF-κB activation through direct regulation of the IKK complex and MAPKs, using both cytoplasmic and nuclear fractions. Such mechanistic validation will be essential to fully delineate the signaling cascade by which CL-80 and ar-turmerone exert neuroprotective effects in the context of Aβ pathology.

Functionally, the anti-inflammatory effects of CL-80 and ar-turmerone translated into significant cognitive improvements in Aβ_1-42_-injected mice. In the Morris water maze, both compounds significantly reduced escape latency and increased the time spent in the target quadrant, indicating improved spatial learning and memory retention [40, 65]. The passive avoidance test further confirmed long-term memory improvement [66]. Notably, the cognitive-enhancing effects of CL-80 and ar-turmerone were comparable to those of SFH, a known memory enhancer [67]. These results are consistent with previous *in vitro* studies reporting the neuroprotective effects of ar-turmerone against Aβ-induced neuronal damage. Although the Aβ_1-42_ injection model used in this study recapitulates key aspects of AD-related neurotoxicity, including neuroinflammation and cognitive deficits, it represents an acute and focal insult rather than the chronic, progressive nature of human AD. Moreover, this model does not reproduce tau pathology or the full spectrum of neurodegenerative changes observed in clinical AD. Therefore, while our findings provide important mechanistic insight into the preventive actions of CL-80 and ar-turmerone, further studies using transgenic or age-related AD models will be necessary to fully validate their therapeutic relevance. Importantly, our findings further demonstrate that such protective effects translate into measurable improvements in behavior and cognition in an *in vivo* AD model, highlighting the therapeutic relevance of ar-turmerone under physiologically relevant conditions. It should be noted that the current study employed a preventive protocol, in which CL-80 and ar-turmerone were administered before Aβ exposure. Thus, the observed improvements in cognition and reductions in neuroinflammation primarily reflect the prophylactic potential of these compounds. Further studies are needed to assess their therapeutic efficacy when administered after disease onset.

In addition to reducing inflammation, both CL-80 and ar-turmerone restored ACh levels and suppressed AChE activity, thereby alleviating the cholinergic deficits commonly observed in AD [68, 69]. These results support the hypothesis that CL-80 and ar-turmerone not only attenuate neuroinflammation but also preserve cholinergic neurotransmission, contributing to cognitive restoration.

Taken together, although further experiments are needed to fully determine the specific mechanisms by which CL-80 and ar-turmerone act on Aβ-induced memory impairment in AD models, our data clearly demonstrated that CL-80 and ar-turmerone inhibited neuronal inflammation by Aβ and improved cognitive ability in an AD model. They may prevent the creation of excessive inflammatory factors by regulating the TLR4/NF-κB signal transduction pathway.

## Conclusion

Our results thus provide new insights into the potential role that Aβ and/or TLR4/NF-κB inhibition could play in alleviating the innate immune response associated with AD and suggest a strong basis for future studies in this field. These findings highlight the therapeutic potential of CL-80 and ar-turmerone in mitigating Aβ-induced neuroinflammation and cognitive dysfunction via inhibition of the TLR4/NF-κB pathway. This pleiotropic mechanism of action positions them as promising candidates for phytopharmaceutical development in the treatment of AD. Although further studies are warranted to elucidate the precise upstream signaling targets, such as IKK and MAPKs, our results clearly demonstrate that CL-80 and ar-turmerone suppress Aβ-induced neuroinflammation and improve cognitive function via inhibition of the TLR4/NF-κB signaling pathway. These findings underscore their therapeutic potential as multifunctional phytochemicals for the treatment of AD.

## Figures and Tables

**Table 1:** Dry matter content of the 80% ethanol extract of *Curcuma longa* (CL-80).

Name of the extract	Dry matter (%)
	80% Ethanol extract 75 °C
CL-80	13.6 %

The dry matter content of the lyophilized extract of *Curcuma longa* prepared using 80% ethanol was determined by drying at 75°C to a constant weight. The result is expressed as a percentage of dry residue based on total extract volume.

**Table 2:** Primer sequences used for quantitative real-time PCR analysis.

Gene	Sense (F)	Anti-sense (R)	Tm (°C)
TLR4 NM_021297	GACTATGTGATGTGACCATT	AGATACACCTGCCAGAGAC	60
NF-κB AJ_002424	GCTCGGCTGAATGAATCTAC	GTGTGGGTGCTTGATGTAAA	60
GAPDH XM_017593963	CAACGGCACAGTCAAGGCTGAGA	CTCAGCACCAGCATCACCCCA	60

Sense (forward) and antisense (reverse) primer sequences were designed and validated for the amplification of target genes using quantitative real-time polymerase chain reaction. Primer specificity was confirmed by melting curve analysis. All sequences are listed in 5' to 3' orientation.
